# Improved Sensitivity in Hydrophilic Interaction Liquid Chromatography-Electrospray-Mass Spectrometry after Removal of Sodium and Potassium Ions from Biological Samples

**DOI:** 10.3390/metabo11030170

**Published:** 2021-03-15

**Authors:** Ida Erngren, Marika Nestor, Curt Pettersson, Mikael Hedeland

**Affiliations:** 1 Analytical Pharmaceutical Chemistry, Department of Medicinal Chemistry, Uppsala University, 75123 Uppsala, Sweden; curt.pettersson@ilk.uu.se (C.P.); mikael.hedeland@ilk.uu.se (M.H.); 2 Department of Immunology, Genetics and Pathology, Uppsala University, 75123 Uppsala, Sweden; marika.nestor@igp.uu.se

**Keywords:** hydrophilic interaction liquid chromatography, mass spectrometry, metabolomics, sample preparation, ion suppression, matrix effects, alkali metal ions

## Abstract

Inorganic ions, such as sodium and potassium, are present in all biological matrices and are sometimes also added during sample preparation. However, these inorganic ions are known to hamper electrospray ionization -mass spectrometry (ESI-MS) applications, especially in hydrophilic interaction liquid chromatography (HILIC) where they are retained and can be detected as adducts and clusters with mobile phase components or analytes. The retention of inorganic ions leads to co-elution with analytes and as a result ion-suppression, extensive adduct formation and problems with reproducibility. In the presented work, a sample preparation method using cation exchange solid phase extraction (SPE) was developed to trap Na^+^ and K^+^ ions from human blood plasma and head and neck cancer cells for the analysis of small cationic, anionic as well as neutral organic analytes. The investigated analytes were small, hydrophilic compounds typically in focus in metabolomics studies. The samples were analyzed using full-scan HILIC-ESI-quadrupole time of flight (QTOF)-MS with an untargeted, screening approach. Method performance was evaluated using multivariate data analysis as well as relative quantifications, spiking of standards to evaluate linearity of response and post-column infusion to study ion-suppression. In blood plasma, the reduction of sodium and potassium ion concentration resulted in improved sensitivity increased signal intensity for 19 out of 28 investigated analytes, improved linearity of response, reduced ion-suppression and reduced cluster formation as well as adduct formation. Thus, the presented method has significant potential to improve data quality in metabolomics studies.

## 1. Introduction

Inorganic alkali metal ions, e.g., sodium and potassium ions, are present in all biological matrices and are sometimes also added during sample processing and preparation. These ions cause a number of issues in electrospray ionization- mass spectrometry (ESI-MS) and can severely hamper the analysis [1,2,3,4,5,6]. The use of reversed phase (RP) liquid chromatography (LC) prior to MS detection often mitigates some of the problems caused by non-volatile inorganic ions, as they generally elute with the void volume. However, when using hydrophilic interaction liquid chromatography (HILIC) the inorganic ions are retained in the chromatographic system and can be detected as cluster ions with mobile phase additives, other inorganic ions, endogenous components of the sample including analytes [2,5,7]. The retention of inorganic ions in HILIC analyses have been studied and utilized for the analysis of counter ions in pharmaceutical formulations, particularly using zwitterionic stationary phases where buffer concentration as well as the organic content of the mobile phase have been concluded to the most important factors to adjust the retention and selectivity of ionic species [8,9]. Different HILIC stationary phases have different selectivities towards inorganic ions which was investigated by Elmsjö et al. [5]; they found that the amide and sulfobetaine stationary phases were preferred for untargeted metabolomics analyses as the retention time range of the inorganic ions were narrower as compared to the bare silica phase meaning that their impact on co-eluting metabolites could be minimized. The co-elution with analytes affects adduct formation, ion-suppression, and thereby sensitivity and reproducibility. McMillan et al. [10] reported that as many as 28% of the features (*m/z*—retention time pairs) in a metabolomics data set were in fact salt clusters and that the proportion of those in the datasets were generally higher using HILIC than RP. The issues caused by inorganic ions have been investigated in targeted quantitative LC-MS/MS applications as well as in untargeted/screening applications e.g., metabolomics [2,5,7]. However, solutions to these problems have not yet been introduced or investigated.

Most sample preparation techniques used in bioanalysis of organic compounds focus on isolating the analytes from components that cause matrix effects. Often liquid-liquid extraction (LLE) or RP- solid phase extraction (SPE) is employed to isolate the analytes and to desalt the samples. However, when the analytes of interest are polar and cannot be extracted by LLE, alternatively not be retained in RP-SPE or mixed-mode SPE, sample preparation becomes a challenge. Johnson et al. [11] used a polymer based mixed-mode column to extract polar organic analytes in sea water and to desalt the samples, however, polar amino acids, sugars and nucleotides were not sufficiently retained on the SPE solid phase for extraction. This type of difficulties often lead to the use of minimal pretreatment such as dilute-and-shoot or protein precipitation (PPT). In some applications it is common to use LLE to separate the lipophilic components from the polar analytes with subsequent analysis of the aqueous phase. However, this leads to high concentrations of inorganic ions in the sample and they are sometimes enriched in the final sample [12].

Some effort has been made to selectively remove Na^+^ and K^+^ prior to analysis of polar organic analytes in complex matrices. Oikawa et al. [13] developed a cation exchange SPE method for the analysis of positively charged amino acids and similar substances in soil samples, where they were able to trap the positively charged inorganic ions on the solid phase and selectively elute the analytes for CE-MS analysis. Whiley et al. [14] used both a cation exchange resin and an anion exchange resin to desalt large samples of pooled urine for preparative LC and downstream identification of urinary metabolites, however, there was no systematic investigation of the analytical performance or if all analytes were eluted from the ion exchange resins. Electromembrane extraction (EME) has been used to separate analytes such as pharmaceuticals and amino acids from inorganic ions as well as phospholipids in biological matrices, but EME requires different system setups for cationic, anionic and neutral analytes and may be cumbersome when a wide range of analytes is of interest [15,16,17]. To the authors’ knowledge, there are no methods that aim to selectively remove inorganic alkali ions prior to bioanalysis of a wide range of small polar analytes that are neutral, positively as well as negatively charged for e.g. metabolomics applications.

In a previous project we investigated and demonstrated the negative effects of alkali metal ions in untargeted HILIC-ESI-MS analyses [7]. The ions caused extensive adduct and cluster formation of analytes, especially of those co-eluting with the inorganic ion. Furthermore, the inorganic ions cause ion-suppression and can affect the quantitative response of analytes. As a continuation of that work, we therefore in this study aimed at developing a sample preparation method to reduce the negative effects of Na^+^/K^+^, in biological samples, i.e., to reduce ion-suppression, reduce cluster formation of analytes with Na^+^/K^+^ and thereby to enhance the analyte sensitivity. Cation exchange SPE was used to trap and remove Na^+^ and K^+^ ions and thereby improve the HILIC-ESI-MS analysis of a wide range of endogenous polar analytes (cationic, anionic and neutral) in biological matrices. The method was developed for untargeted screening applications, such as metabolomics. Evaluation of the method included a wide selection of endogenous metabolites and for a further untargeted evaluation multivariate data analysis was performed. Standard solutions, human plasma as well as head and neck cancer cell extracts were used for the evaluation. Sensitivity and variability in response of the analytes, ion-suppression, adduct and cluster formation as well as linearity of response were investigated and compared to those of generic sample preparation protocols.

## 2. Results and Discussion

Inorganic alkali metal ions are present in all biological matrices and cause numerous issues in HILIC-ESI-MS applications, e.g., ion-suppression, adduct and cluster formation, thereby affecting detection sensitivity [7]. In applications where the analytes of interest are very hydrophilic, e.g., endogenous low molecular weight compounds, the inorganic ions are often isolated along with the analytes and sometimes enriched during sample preparation. In this study, cation exchange SPE was tested for trapping of Na^+^ and K^+^ on the solid phase while eluting the analytes. First, four different cation exchange cartridges were screened for their ability to trap Na^+^ as well as the increase or decrease in signal intensity of a set of selected analytes in standard solution. From the initial experiments the Oasis MCX and Oasis WCX cartridges performed the best. Moving on the two cartridges were tested using human plasma, where it was investigated if controlling the pH in all extraction steps would be beneficial as well as if protein precipitation was necessary prior to SPE. Finally the Oasis MCX cartridge was used on a larger set of human plasma samples as well as head and neck cancer cells to evaluate the performance of the final SPE method. The sample preparation method was developed for untargeted screening methods such as metabolomics. Therefore, a wide selection of polar endogenous metabolites from different metabolite classes, with different functional groups and with different physicochemical properties such as log P, pKa and molecular weight was investigated. Detailed information on the investigated metabolites in standard solution, human plasma and cancer cell extracts can be found in the Supplementary Data (Supplementary Data Tables S1–S3).

Multivariate data analysis was used to compare only PPT to PPT+SPE in plasma samples for an untargeted investigation of the metabolites gaining or losing in sensitivity using the SPE protocol. The results were evaluated with emphasis on sensitivity of detection and signal intensity, ion suppression, adduct formation as well as variability in response and linearity of response of endogenous analytes. The experiments were performed in standard solutions, human blood plasma as well as in head and neck cancer cells. The developed SPE protocol using Oasis MCX cartridges to reduce the Na^+^ and K^+^ concentration was compared to methods generally used in metabolomics, protein precipitation in the case of human plasma and to LLE in the case of cancer cell extracts.

### 2.1. Development of the SPE Protocol

Three different strong cation exchange solid phase cartridges (Isolute SCX-2, StrataX-C, Oasis MCX) and one weak cation exchanger (Oasis WCX) were tested for their ability to remove Na^+^ from an aqueous standard solution containing 120 mM NaCl, furthermore the relative peak area of 15 selected analytes as compared to neat solution was evaluated (see Section 3.2 for a list of analytes and Supplementary Data, Table S1). The Oasis WCX cartridges were tested with two different protocols, one with an acidic elution using 0.0125% formic acid in MeOH followed by 0.0125% formic acid in 1:1 MeOH:water (*v*/*v*) (denoted WCX acidic) and one with the same elution medium as for the other protocols, 0.0125% ammonium hydroxide in MeOH followed by 0.0125% ammonium hydroxide in 1:1 MeOH/water (*v*/*v*) (denoted WCX basic). Two different elution media were evaluated only for Oasis WCX, since this was the only tested weak cation exchanger (carboxylic acid-based), for which a change in pH would have a greater impact on analyte retention than for the strong cation exchangers. The analytes were chosen from different endogenous substance classes, to span over the entire HILIC retention time range, e.g., carboxylic acids, amines, amino acids and quaternary ammonium compounds (Supplementary Data, Table S1). The Oasis MCX protocol performed overall best in these experiments. The peak area of the Na^+^ cluster (Na^+^(HCOO^-^Na^+^)_n_) was reduced by 95.7% in the samples prepared by the SPE MCX protocol and 11 out of 15 analytes had a relative peak area above 100% (average peak area in SPE treated samples/average peak area in standard solution) (Appendix A, Table A1). Moreover, this one resulted in the overall lowest variation between replicates. The WCX basic protocol was the protocol that gave the highest relative peak areas, and 14 out of 15 analytes had a relative peak area above 100%. However, it was not as effective in reducing the Na^+^ concentration and the CV (%) of peak areas between replicates was higher than for the MCX protocol (Appendix A, Table A1). It was hypothesized that the WCX protocol would benefit from pH buffering and that the poor repeatability observed in the initial experiments would improve if pH was controlled during extraction. Therefore the MCX as well as the WCX basic protocols were tested with and without pH buffering (pH = 8) in all extraction steps on human blood plasma. Plasma samples that had gone through only protein precipitation (PPT) as well as protein precipitated plasma prepared with the previous protocols without pH buffering were used as references. No improvement in relative peak area or repeatability was observed by pH buffering for neither the MCX nor the WCX protocols (Appendix A, Table A2). The most suitable protocol was found to be the MCX protocol without pH buffering. Consequently, all further experiments were performed with MCX. It was also concluded that protein precipitation prior to SPE was necessary in order to improve repeatability and to avoid clogging of the cartridges (Supplementary Data, Table S6).

### 2.2. Evaluation of the Final SPE Protocol in Plasma Samples

The final MCX protocol was then tested on a set of pooled human plasma samples and compared to samples only treated with protein precipitation (PPT). Ten identical samples were prepared with the respective treatment procedures (SPE MCX and PPT). The QC sample was injected every fifth injection to ensure stable conditions through-out the analysis. Out of the 28 investigated analytes, the peak area for 25 of them had a CV below 10%, all had a CV below 30%, and the maximum retention time drift was 0.04 min in the QC samples.

#### 2.2.1. Improved Sensitivity of Hydrophilic Analytes by Reduction in K^+^ and Na^+^ Concentrations through SPE

**Figure 1:**
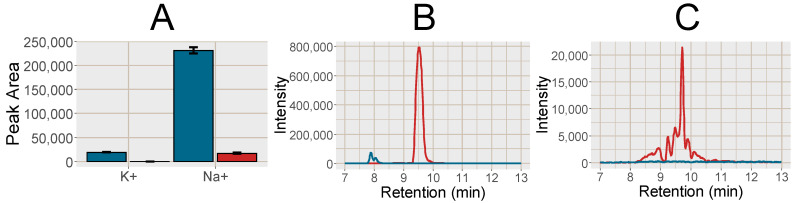
(**A**) Bar plot over the peak area of K^+^ detected as K^+^(HCOOK), *m*/*z* 122.926 and Na^+^ detected as Na^+^(HCOONa)_3_), *m*/*z* 226.954 in the PPT (blue) and SPE MCX (red), error bars show the standard deviation (n = 10). (**B**) Chromatogram of K^+^ (blue line) and Na^+^ (red line) in the PPT. (**C**) Chromatogram of K^+^ (blue line) and Na^+^ (red line) in the SPE MCX.

The response of 28 endogenous and food related analytes were investigated in the plasma samples (for a list of substances see, Supplementary Data, Table S3). In the MCX SPE samples compared to PPT, the Na^+^ cluster peak area was reduced by 92.8% and the K^+^ cluster peak was reduced to below the detection limit (Figure 1). An important observation from Figure 1B,C is the width of the Na^+^ peak, evident in especially Figure 1C due to the different scale of the y-axis. This leads to effects on co-eluting analytes in a large retention time window, which is further discussed in more detail below (Section 2.2.2).

**Figure 2:**
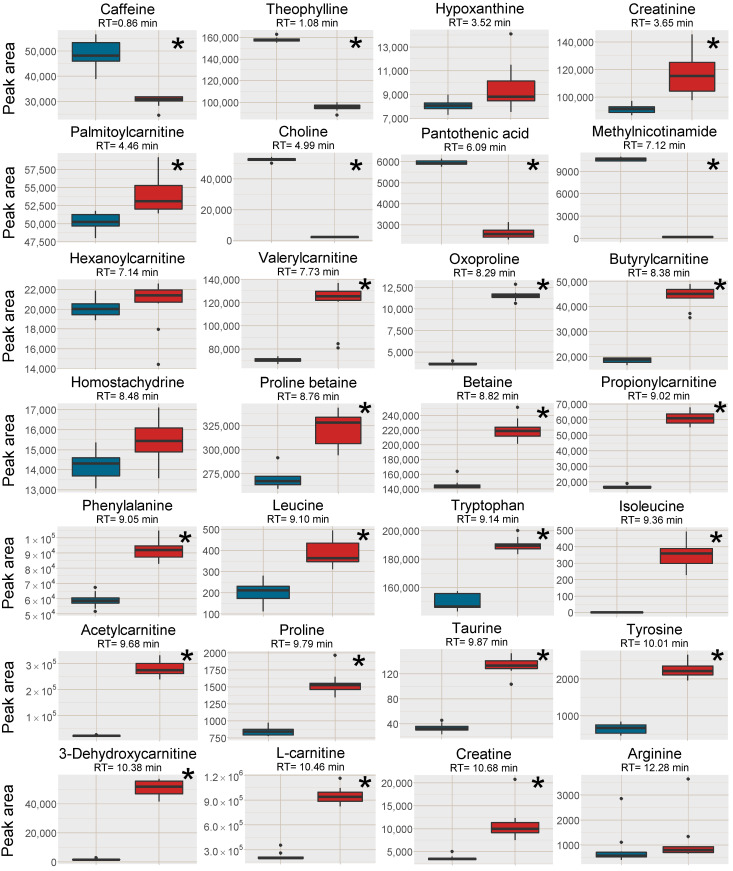
Box plots of the peak area of [M+H]^+^ (or [M ]^+^ where applicable) of all investigated analytes in the plasma samples, red box=SPE MCX, blue box = PPT. The outer edges of the boxes represent the first and third quartiles respectively while the middle line represent the median and outliers are denoted by dots in the plots. Analytes with statistically different peak area (*p* < 0.05 in a unpaired *t*-test with bonferroni correction applied) between the two sample treatments are marked with a * in the top right corner of each individual box-plot.

Increased signal intensity for [M+H]^+^ was observed in the SPE MCX samples for 22 out of the investigated 28 analytes, two were not affected and five analytes signal intensity was reduced (Figure 2). A majority of the analytes with increased signal intensity eluted between 7 and 12 min, i.e., co-eluting with or eluting in close proximity to Na^+^ and K^+^ clusters (Figure 1 and Figure 2). Mainly polar analytes such as amino acids and short-chain acyl carnitines were among the analytes with increased signal intensity using MCX SPE. Isoleucine could not be detected as [M+H]^+^ at all in the PPT samples but could be detected well above the detection limit in the SPE MCX samples. Detection of the [M+H]^+^ adduct is very valuable as the Na^+^ adducts can suffer from poor reproducibility, especially if the Na^+^ concentration differ between samples [7].

Five of the investigated analytes lost in signal intensity using the SPE MCX sample preparation, i.e. caffeine, theophylline, choline, pantothenic acid and 1-methylnicotinamide. Choline and 1-methylnicotinamide are both quaternary ammonium compounds with high affinity for the negatively charged MCX solid phase and were probably not efficiently eluted from the cartridges. This was confirmed by collecting each fraction separately during sample preparation, i.e., the protein precipitated plasma sample filtered through the SPE cartridge, 1st eluate using 0.0125% NH_4_OH in MeOH, 2nd eluate using 0.0125% NH_4_OH in 1:1 MeOH:water (*v*/*v*) and an extra 3rd elution step using 0.6 M ammonium formate in water at pH = 4. Both these analytes were found in very high intensity in the 3rd elution step (Supplementary Data, Figure S1). However, this extra step also eluted the Na^+^ and K^+^ ions, and would not be suitable to use. Pantothenic acid was found in very low amounts in all fractions, however, it suffers from low stability in both acidic and alkaline solutions and have most likely degraded during SPE extraction [18]. It should be stressed that the five analytes with lower intensity in the SPE MCX samples, were still detectable at sufficient intensity for relative quantification in metabolomics applications.

In contrast, quaternary ammonium compounds with carboxylic acid moieties gained in signal intensity using the SPE MCX preparation (acylcarnitines, betaine and betaine derivatives) probably because the elution at pH 8 increased their negative charge and thereby reduced their affinity for the solid phase. However, strong retention to the solid phase was not the reason for the reduction in signal intensity of caffeine and theophylline. For both compounds, the highest intensity was found in the combined extract and in the 1st eluate of the separately analyzed fractions (Supplementary Data, Figure S1). During multivariate data analysis (discussed in Section 2.2.3) plasticizers originating from the SPE cartridges were found to elute during the first 2 min, which could indicate that the loss in signal intensity was due to increased ion-suppression in the beginning of the chromatogram.

**Figure 3:**
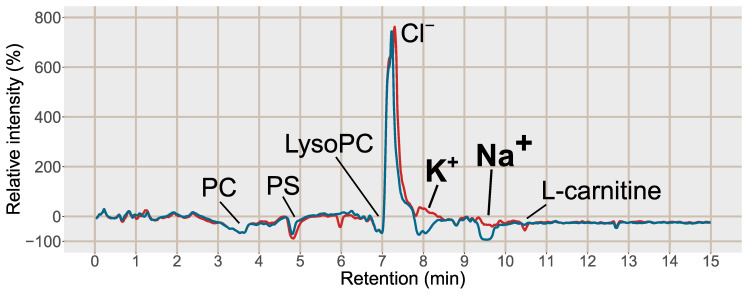
Post-column infusion chromatogram of guanine (2*^13^C, ^15^N) with the relative intensity in % as compared to the injection of a solvent blank on the y-axis 100*(Intensity_MCX or PPT_/Intensity_blank_) − 100. Blue line = injection of blood plasma treated with PPT, red line = injection of blood plasma treated with SPE MCX. Each line is based on the average of 3 samples of each type, PPT, SPE MCX or a solvent blank. Major suppressions/enhancements are annotated in the chromatogram, PC = phosphatidylcholines, PS = phosphatidylserines, LysoPC = Lysophosphatidylcholines, K^+^ = potassium formate cluster K^+^(HCOOK), *m*/*z* 122.926, Na^+^ = sodium formate cluster Na^+^(HCOONa)_3_), *m*/*z* 226.954, Cl^−^ = potassium chloride cluster, K^+^(KCl), *m*/*z* 112.8963.

Post-column infusion experiments were carried out to investigate the ion-suppression as well as adduct formation throughout the chromatogram [19]. Isotopically labelled analogues of guanine (^13^C, 2*^15^N), hypoxanthine (3*D), inosine (4*^15^N), tryptophan (5*D), valine (8*D) and glutamine (5*D) were infused post-column and plasma samples prepared by PPT or SPE MCX were injected. The isotopically labelled analogues were selected as they are not naturally present in the plasma, however, they can be expected to ionize as their non-labelled analogues. One reason behind the increase in signal intensity for [M+H]^+^ shown in Figure 2 was reduced ion-suppression from the formate clusters of Na^+^ and K^+^ observed as disappearing negative peaks at 8.0 and 9.5 min, respectively, using SPE MCX (Figure 3). In the PPT samples the ion-suppression from the Na^+^ cluster lead to almost complete extinction of the analyte signal (−100%). There was also a large enhancement at 7.5 min from the chloride cluster ([K^+^(KCl)_n_]^+^) that was unaffected by the type of sample preparation method. Hence a major reason for the increased signal intensity of the analytes in the SPE treated sample were reduced ion-suppression from the inorganic ion clusters. 

#### 2.2.2. Decreased Adduct and Cluster Formation Due to Reduced Na^+^ and K^+^ Concentrations

**Figure 4:**
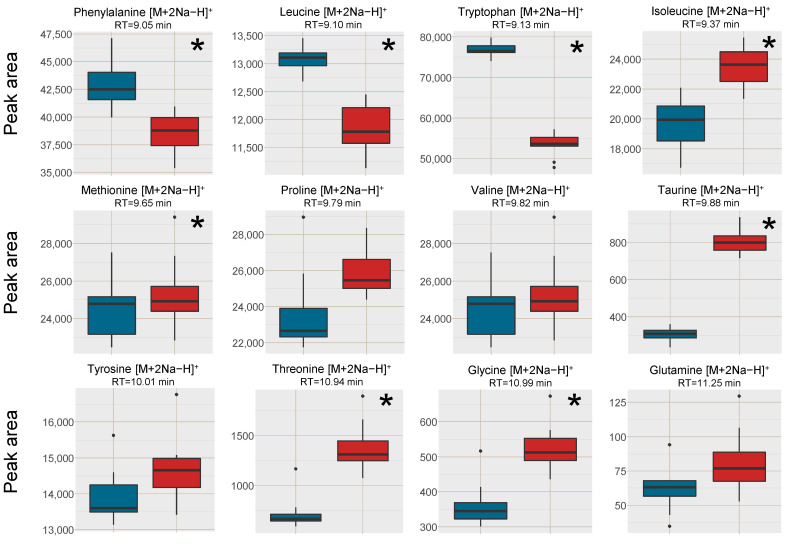
Box plots of the peak area of [M+2Na−H]^+^ of amino acids in human blood plasma samples, red box = SPE MCX, blue box = PPT. The outer edges of the boxes represent the first and third quartiles respectively while the middle line represent the median and outliers are denoted by dots in the plots. Analytes with statistically different peak area (*p* < 0.05 in a unpaired *t*-test with bonferroni correction applied) between the two sample treatments are marked with a * in the top right corner of each individual box-plot.

**Figure 5:**
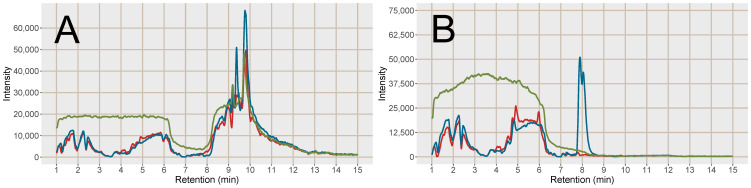
Post-column infusion chromatograms of tryptophan (5*D) Na^+^/K^+^ adducts, (**A**) [M+2K−H]^+^ and (**B**) [M+2Na−H]^+^. Red line = injection of blood plasma sample prepared with SPE MCX, blue line = injection of blood plasma sample prepared with PPT and green line = injection of a solvent blank. Each line is based on the average of 3 samples of each type, PPT, SPE MCX or blank.

A second reason for the increased intensity of the [M+H]^+^ ions using MCX compared to only PPT, was reduced alkali metal ion adduct formation as well as reduced cluster formation of analytes (with Na^+^/K^+^ and formate), leading to fewer signals per analyte. This was investigated through the adduct formation of amino acids in the plasma samples (Figure 4) as well as in post-column infusion experiments (Figure 5), where the adduct formation of the infused analytes can be traced over the elution gradient. 

Regarding the formation of [M+2Na−H]^+^ adducts of amino acids from the two sample preparation methods, the intensity was higher in the PPT samples for phenylalanine, leucine and tryptophan, which were the three earliest eluting and the least polar amino acids (Figure 4). The later eluting amino acids, eluting after the Na^+^ cluster were however found in higher intensities (Na^+^ adducts) in the SPE MCX samples than in the PPT samples. This could indicate that it was not necessarily only the availability of Na^+^ ions that was the limiting factor for the adduct formation but rather a combination of availability of Na^+^ and ion-suppression from Na^+^. Bonfiglio et al. [19] reported that polar analytes were more susceptible to ion-suppression, which could explain why both Na^+^ adducts and [M+H]^+^ of the amino acids eluting after the Na^+^ cluster were increased in intensity in the SPE MCX samples. However, phenylalanine, leucine and tryptophan might be less susceptible to ion-suppression, resulting in high intensity of the Na^+^ adducts due to the extremely high availability of Na^+^ ions in the PPT samples.

#### 2.2.3. Multivariate Data Analysis

**Figure 6:**
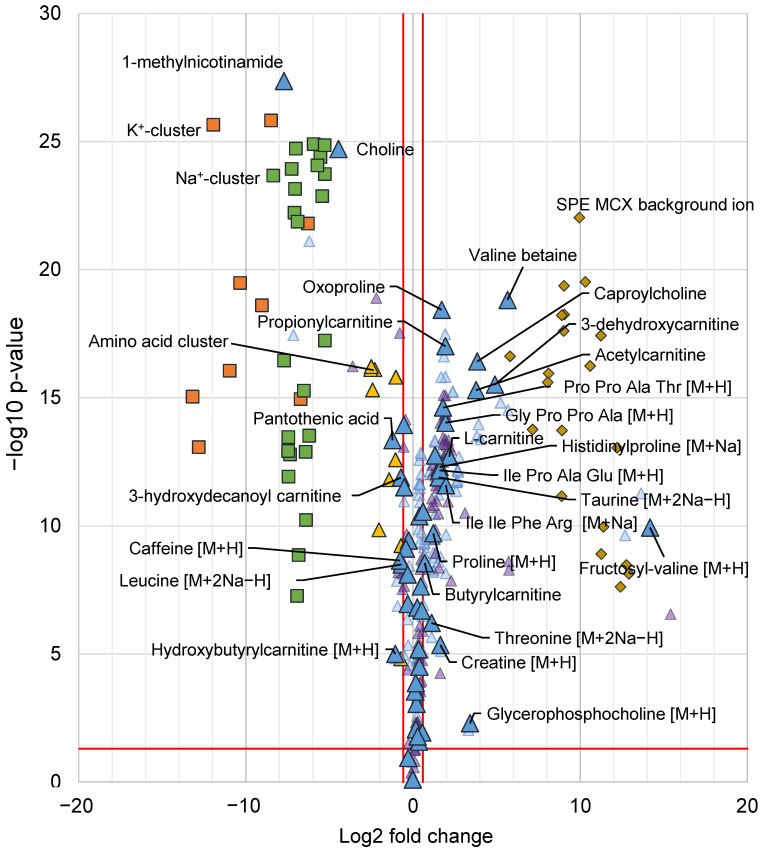
Volcano plot with all annotated features from the multivariate data analysis, indicated as differentiating between the SPE MCX and PPT samples. X-axis shows log2 fold change and the y-axis -log10 *p*-value from a unpaired *t*-test. A positive log2 fold change indicates higher intensity in the SPE MCX samples and negative log2 fold change indicates a higher intensity in PPT samples. Metabolites are denoted by triangles, for each annotated metabolite the [M+H]^+^ or corresponding signal is denoted by a large blue triangle (depending on which ions were detected), fragment ions or isotopes are denoted by smaller light blue triangles, adducts are denoted by small purple triangles. All metabolites with a fold change corresponding to 1.5 or more and a *p*-value < 0.05 (indicated by red lines) are labelled with their respective annotation. K^+^ clusters (K^+^(HCOOK)_n_) denoted by orange squares (■), Na^+^ clusters (Na^+^(HCOONa)_n_) are denoted by green squares (■), clusters containing amino acids are denoted by yellow triangles (▲). Background ions from the SPE cartridges are denoted by dark yellow diamonds (♦). All annotated features can be found in Supplementary Data Tables S5 and S6.

Multivariate data analysis was applied as an untargeted approach to study the differences between the two sample preparation methods (MCX SPE and PPT). The signal intensities of analytes as well as patterns of adduct and cluster formation were studied using PCA and OPLS-DA. Features (*m/z*—retention time pairs) that were indicated as important for the differences between the two methods were annotated. The differentiating features are summarized in a volcano-plot (Figure 6), where the log2 fold change (X-axis) are plotted against the -log10 *p*-value from an unpaired *t*-test. In the volcano plot only analytes with a fold change larger than 1.5 and *p*-value lower than 0.05 was labelled and furthermore, only one feature per analyte was labelled as most analytes were represented by multiple features. All annotated features per analyte is summarized in the Supplementary Data Tables S5 and S6.

Among the analytes with higher intensity in the PPT samples were the already described and discussed, 1-methylnicotinamide, choline, pantothenic acid and caffeine (see Section 2.2.1), moreover, two acylcarnitines were also found in higher intensity in the PPT samples as well as the sodium adduct [M+2Na−H]^+^ of leucine. A few analytes were found in higher intensity in the PPT samples but just below the 1.5-fold change limit among those were six acylcarnitines as well as theophylline found. A large number (18) of analytes were found in higher intensity (fold change > 1.5) in the SPE MCX samples, a majority of those metabolites were amino acids and derivatives thereof as well as carnitines and smaller peptides (up to four amino acids). There were some metabolites that were found just below the fold change limit of fold change 1.5, among those were nine amino acids found, three acylcarnitines and hypoxanthine (Figure 6). Acylcarnitines were found with higher intensity in both sample groups, in the SPE MCX samples, mainly short-chain acylcarnitines, eluting after 7 min gained in signal intensity, whereas in the PPT samples mainly long-chain acyl carnitines, which elute earlier (retention 5–6 min) had a higher signal intensity. This is in line with the previous discussion on the type of analytes that were affected by the reduced alkali ion concentration. In the MCX samples, a total of 16 different plasticizers (represented by 20 features in Figure 6) and other types of background ions not present in the PPT samples, but also present in extraction blanks from the SPE cartridges, were picked up by the multivariate models. This highlights a drawback with SPE as it has been shown to give higher background and add possible ion-suppressors to the samples [20]. A majority of the background ions from the SPE MCX eluted in the first 2 min of the chromatogram, which could explain the loss in signal intensity for the early eluting caffeine and theophylline (Figure 2) but it also highlights the benefit of the SPE MCX for the analysis of late eluting analytes. Despite the background of the SPE MCX samples, there was a major gain in signal intensity for a number of analytes, while the loss in signal intensity (higher signal intensity in PPT) was very limited. In the SPE MCX samples, 31 highly relevant analytes (18 with fold change > 1.5 and 13 with fold change < 1.5) central in human metabolism were found in higher intensity, while in the PPT samples only 13 relevant analytes were found in higher intensity. However, it should be stressed that the analytes with higher intensity after PPT, were still detectable in the SPE MCX samples and could be used for relative quantifications.

The impact of the reduction in alkali metal ion concentration in the samples treated with SPE MCX was further emphasized as the distribution of adducts and clusters were investigated among all the annotated features (Figure 6). The reduction in cluster formation of alkali ions, as well as cluster formation of amino acids using SPE MCX, stood out as one of the major differences between the two sample preparation methods and most cluster ions were not found at all in the SPE MCX samples. A majority of the annotated features with higher intensity in the PPT samples (or features that were only found in the PPT samples) were cluster ions or adducts with Na^+^ or K^+^, whereas for the features with higher intensity in the MCX samples the majority were [M+H]^+^ or fragment ions [F]^+^. The differences in adduct formation and cluster formation between the two sample preparations highlights the reduction in data complexity using the SPE MCX sample preparation. Excessive adduct and cluster formation inflate the dataset leading to multiple features per analyte, which hampers data interpretation as well as analyte identification.

In conclusion, the signal intensity was improved for a majority of the investigated analytes using the SPE MCX sample preparation method. The gain in signal intensity was an effect of the reduced ion suppression, but also due to reduced adduct formation and reduced cluster formation of analytes. The sensitivity of a few analytes was reduced with the SPE MCX sample preparation, however they were still detectable with sufficient intensity for relative quantification in e.g. metabolomics applications. Furthermore, the results highlight multivariate data analysis as an important tool to find patterns in the entire dataset regarding signal intensities as well as adduct formation and cluster formation.

#### 2.2.4. Linear Response of Spiked Analytes Were Improved

In metabolomics analyses, linear response of analytes is often assumed but rarely investigated. As the SPE MCX method had large effects on the ion-suppression and adduct formation, it could also have effects on the linearity of response of analytes, for the better or for the worse depending on reproducibility in reduction in Na^+^ and K^+^ concentration. Therefore the linearity of response of four isotopically labelled standards, guanine (^13^C, 2*^15^N), inosine (4*^15^N), tryptophan (5*D indole) and valine (8*D) was investigated and compared between SPE MCX and PPT. The standards were spiked in plasma at eight different concentrations and prepared with SPE MCX or PPT (3 replicates per concentration). The selected standards were all hydrophilic analytes that eluted in the proximity to the alkali metal ion clusters and should be affected by the reduction in Na^+^/K^+^ concentration. The results were evaluated based on the number of standards within ±15% of their nominal concentrations and the concentration range of the accepted standards (Table 1). The tested concentration ranges were selected to include the expected concentrations in blood plasma for each analyte. The [M+H]^+^ adduct or the main fragment [F]^+^ in case of complete in-source fragmentation, were investigated. 

Since many of the small polar amino acids can be difficult to detect as their respective [M+H]^+^ ions (or fragments), but are easily detected as [M+2Na−H]^+^, the linearity of the alkali adducts were investigated for valine and tryptophan as well. For valine [M+2Na−H]^+^ the results were similar with both sample preparation methods with good linearity over the entire tested range. However, for tryptophan no linear range could be determined in the PPT samples, and in the MCX samples only five samples were within ±15% of the nominal values. In both calibration curves, there was a curvature towards saturation in the higher concentration range (data not shown), which was more pronounced in the PPT samples. This could be explained by more a pronounced ion-suppression in the PPT as compared to the SPE MCX samples. In Figure 4 there were large differences in the responses of the sodium adducts between valine and tryptophan when comparing the two sample preparations. Valine’s adduct response was not significantly different between PPT and SPE MCX, whereas the response of the tryptophan sodium adduct was much higher in the PPT samples. This could explain the issues with saturation effects in the tryptophan calibration curve and the differences between valine’s and tryptophan’s linearity in response. For relative quantitative purposes the results suggest that sodium adducts as quantitative target ions should be used with caution, as the response is not necessarily linear and as reported previously the adduct response can differ depending on the individual alkali ion concentration of the sample [7]. 

However, if the aim of the application is to simply determine up/down regulations between sample groups and not the exact magnitude of change, then the linearity issues described here should not be a problem. In conclusion, the linearity of response was improved or unchanged for the tested analytes using the SPE MCX sample preparation. The main reason behind the improvement seemed to be the reduced ion-suppression in the SPE MCX samples, leading to higher sensitivity and less variation between replicates in the lower concentration ranges as well as less saturation effects in the higher concentration ranges.

### 2.3. Cell Samples 

**Figure 7:**
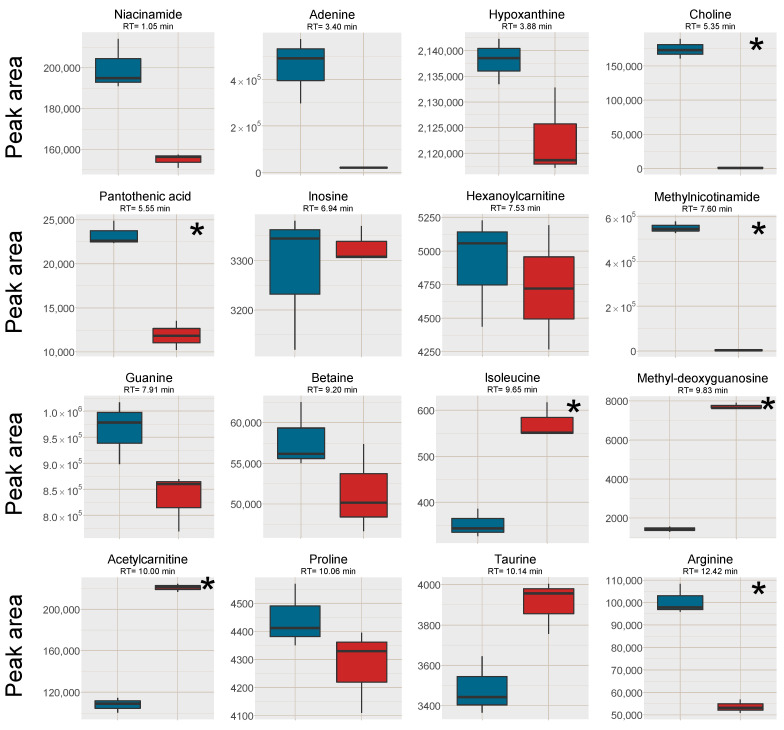
Box plots of the peak area of [M+H]^+^ (or [M]^+^ where applicable) of all investigated analytes in the cell samples, red box = SPE MCX, blue box = LLE. The outer edges of the boxes represent the first and third quartiles respectively while the middle line represent the median and outliers are denoted by dots in the plots. Analytes with statistically different peak area (*p* < 0.05 in a unpaired *t*-test with bonferroni correction applied) between the two sample treatments are marked with a * in the top right corner of each individual box-plot.

To test the SPE MCX sample preparation on another matrix than the pooled human plasma, head and neck cancer cell extracts were pooled and divided into 6 aliquots. The samples were then subjected to LLE for removal of lipophilic compounds and the aqueous phases were collected [21,22]. Out of the six samples, three were further prepared with the same SPE MCX method as the plasma samples. Analytes (n = 16) that spanned the entire retention time range, of different substance classes and physicochemical properties were investigated with regard to signal intensity and the removal of Na^+^ and K^+^ was investigated as well (Supplementary Data, Table S3). The Na^+^ and K^+^ peaks were more well-defined and narrow in the cell samples after both pretreatment methods, i.e. they did not affect as large retention time window as in the plasma samples. The peak areas were also slightly smaller than in the plasma samples suggesting a lower alkali metal ion concentration in the cell samples. Lower Na^+^ and K^+^ concentrations could explain the differences in results between the plasma samples and the cell samples (Figure 7, cf. Figure 2). The strongest positive effects of SPE on signal intensity were for the analytes co-eluting with the Na^+^ cluster: isoleucine, methyldeoxyguanosine and acetylcarnitine, whereas the results for most of the other analytes favored the LLE sample preparation without further SPE. This was likely because ion-suppression from Na^+^ and K^+^ was not as detrimental to the results of the cell samples as was observed in the plasma samples and that fewer analytes in cell samples benefitted from the reduction of K^+^ and Na^+^.

## 3. Material and Methods

### 3.1. Chemicals

Acetonitrile, methanol, formic acid and ammonium formate, all LC-MS grade were purchased from Fisher Scientific (Loughborough, UK). Ammonium hydroxide (25%, LC-MS grade) was bought from Merck (Darmstadt, Germany) and chloroform (HPLC-grade) was bought from BDH chemicals (Poole, UK). The isotopically labeled standards, guanine (^13^C, 2*^15^N, 98%), hypoxanthine (3*D, 98%), inosine (4*^15^N, >95%), tryptophan (5*D, 98%) and glutamine (5*D, 97%) were purchased from Cambridge Isotope laboratories (Andover, MA, USA) while valine (8* D, 98%) was obtained from Aldrich (St Louis, MO, USA). The standard compounds butyrylcarnitine (≥97%), hypoxanthine (≥99%), choline (≥99%), adenine (≥99%), L-tryptophan (≥99.5%), acetylcarnitine (≥99%), L-proline (≥99.5%), L-carnitine (≥98%) and L-arginine (≥98%) were obtained from Sigma (Steinheim, Germany). Phenylalanine was purchased from Merck. Creatine (99.5%) was bought from Chem Service (West Chester, PA, USA) and NADH (>95%) was bought from Appli Chem (Darmstadt, Germany). Betaine (>99%), taurine (>99%) and thiamine (99.9%) were purchased from Fluka (Steinheim, Germany). All water was purified using a Milli-Q purification unit (MilliporeMerck, Darmstadt, Germany).

### 3.2. SPE on Standard Solutios

A standard solution containing 15 analytes at 20 µM (acetylcarnitine, adenine, arginine, betaine, butyrylcarnitine, carnitine, choline, creatine, hypoxanthine, NADH, phenylalanine, proline, taurine, thiamine and tryptophan) as well as 120 mM NaCl was diluted in water. The analytes were selected to contain several different metabolite classes and functional groups as well as span a broad selection of log P, pKa, molecular size and retention times in the used HILIC-ESI-MS system. More detailed information on the selected analytes can be found in the Supplementary Data Table S1. 

Four different SPE cartridges: Oasis MCX (Waters, Milford, MA, USA), Oasis WCX (Waters), StrataX-C (Phenomenex, Torrance, CA, USA) and Isolute SCX-2 (Biotage, Uppsala, Sweden) were evaluated. The strong cation exchange cartridges (Oasis MCX, StrataX-C and Isolute SCX-2) were conditioned and equilibrated with methanol (MeOH) and water consecutively. For all protocols 100 µL of the standard solution was diluted with 300 µL acetonitrile (ACN), to replicate the conditions of a protein precipitation (PPT) and was then added to the equilibrated cartridges. Retained analytes were eluted with 0.0125% ammonium hydroxide in MeOH followed by 0.0125% ammonium hydroxide in 1:1 MeOH:water (*v*/*v*). 

The weak cation exchange cartridge Oasis WCX cartridge was conditioned with MeOH and equilibrated with 0.0025% ammonium hydroxide in MeOH. The samples (100 µL standard solution + 300 µL ACN) were added to the cartridges. The Oasis WCX cartridge was tested with two different protocols. The first with acidic elution (WCX acidic), 0.0125% formic acid in MeOH followed by 0.0125% formic acid in 1:1 MeOH:water (*v*/*v*). The second with basic elution (WCX basic), 0.0125% ammonium hydroxide in MeOH followed by 0.0125% ammonium hydroxide in 1:1 MeOH:water (*v*/*v*). For all protocols the sample that passed through the cartridge (non-retained analytes) and the two eluates were combined and evaporated to dryness in a vacuum concentrator (Eppendorf, Hamburg, Germany) at 30 °C.Aliquots of the standard solution (100 µL) mixed with 300 µL ACN were evaporated as well and used as reference to evaluate the performance of the SPE protocols. All samples were reconstituted just prior to analysis in 100 µL, 1:9 water:ACN (*v*/*v*). 

### 3.3. Final SPE Method and Preparation of Plasma Samples

Pooled drug free human blood plasma with Na-EDTA was purchased from 3H Biomedical, Uppsala, Sweden. The pooled plasma was thawed in room temperature and aliquoted into samples of 100 µL each (*n* = 20). The proteins were precipitated by addition of 300 µL cold ACN to each sample. The samples were vortexed for 15 s and centrifuged at 4 °C and 877× *g* for 20 min. The supernatants (375 µL) were isolated and half of the samples (*n* = 10) were further prepared by cation exchange SPE. The cartridges, Oasis MCX (1 mL, 30 mg, Waters) were conditioned and equilibrated with MeOH and water. The supernatants from the protein precipitation were passed through the cartridges into clean tubes, retained analytes were eluted with 500 µL MeOH + 0.0125% NH_4_OH followed by 500 µL 1:1 MeOH:water (*v*/*v*) + 0.0125% NH_4_OH. The filtrated supernatant as well as the two elution fractions were combined. All samples, the samples prepared with PPT+SPE and the samples prepared with just PPT, were evaporated to dryness in a vacuum concentrator at 30 °C. Three additional samples were prepared by SPE as described above, but each elution fraction was collected separately. To elute strongly retained analytes and inorganic ions an extra elution step was added to the samples where each fraction was collected separately with 0.6 M ammonium formate adjusted to pH = 4 (with formic acid) in water. The samples were reconstituted just prior to analysis in 100 µL 1:9 water:ACN (*v*/*v*). A QC sample was pooled from all samples, both SPE MCX and PPT by taking 10 µL from each reconstituted sample.

### 3.4. Post-Column Infusion Experiments

A solution containing guanine (^13^C, 2*^15^N) 6.6 µM, hypoxanthine (3*D) 8.0 µM, inosine (4*^15^N) 8.0 µM, tryptophan (5*D) 8.0 µM, valine (8*D) 8.0 µM and glutamine (5*D) 8.0 µM, diluted in ACN was infused post-column with a flow rate of 10 µL min^−1^ and mixed in a T-junction with the mobile phase flow. The final concentration in the mobile phase flow was 0.16 µM for guanine (^13^C, 2*^15^N) and 0.20 µM for the rest of the standards. Three replicates of each plasma sample type SPE MCX, PPT and a solvent blank (1:9 water:ACN (*v*/*v*)) were injected during the infusion of the isotopically labeled standard mix. The ion-traces of the [M+H]^+^, [M+Na]^+^, [M+K]^+^, [M+2Na−H]^+^ and [M+2K−H]^+^ adducts of the infused substances were then investigated to compare matrix effects between the sample types. Relative intensity profiles were calculated by taking the average of the three replicates for each sample type and calculating the relative intensity for each compound and adduct as compared to that of the solvent blank: 100*(Intensity_MCX or PPT_/Intensity_blank_) − 100 [19].

### 3.5. Determination of Linearity

Limits of quantification (LOQ) was first estimated for guanine (^13^C, 2*^15^N), inosine (4*^15^N), tryptophan (5*D) and valine (8*D) in neat solution by diluting standard solutions until the peak heights were approximately 10 times the noise level, signal to noise (S/N) ratios were calculated in Targetlynx (Waters). Plasma samples were then spiked at 8 different concentrations, with the lowest concentration at S/N = 10, 3 replicates at each concentration for the respective sample preparations (PPT or SPE MCX) were prepared. The concentration ranges for the respective analytes were 17.9 nM–1.94 µM for guanine (^13^C, 2*^15^N), 1.10 µM–110 µM for tryptophan (5*D), 79.4 nM–5.40 µM for inosine (4*^15^N) and 77.3 µM–309 µM for valine (8*D). The samples were prepared as described in Section 3.3. The linearity of response of each analyte was evaluated based on the R^2^-value of the regression, the number of standards within ± 15% of nominal concentration when back calculating their concentrations using the regression equation as well as the concentration range of the passed standards. For a valid concentration range, at least 5 standards had to be within ±15% of nominal concentration.

### 3.6. Preparation of Cell Samples

The cell lines UM-SCC-74A and UM-SCC-74B from head and neck squamous cell carcinoma, previously described by Brenner et al. [23], were kindly provided by Professor T.E. Carey (University of Michigan, Ann Arbor, MI, USA). In short the cells were adherently grown, cell medium was removed and the cells were washed with phosphate buffered saline, detached using a cell scraper and collected in water where after the cells were snapfrozen in liquid nitrogen with subsequent freeze-thaw cycles and sonication. The cell culture conditions and harvesting have been in described in detail previously [24]. The samples, five from each cell line, were thawed at room temperature, vortexed for 10 s, centrifuged at 877× *g* and 4 °C for 20 min. An aliquot was taken from each sample to a pooled sample that was further divided into 6 samples of 250 µL each. Liquid-liquid extraction was performed by addition of 350 µL MeOH and 500 µL of chloroform, thereafter the samples were vortexed gently for 15 s, left in 8 °C for 20 min and finally centrifuged at 877× *g* at 4 °C for 20 min. The aqueous phases were isolated, and for three of the replicates cation exchange SPE was performed using the same SPE protocol as described above under Section 3.3 however, with a different sample volume (600 µL) and slightly different sample composition. All replicates, three samples treated with LLE only and three samples treated with LLE followed by SPE were evaporated to dryness in a vacuum concentrator at 30 °C. The samples were reconstituted just prior to analysis in 175 µL 1:4 water:ACN (*v*/*v*).

### 3.7. HILIC-QTOF-ESI-MS Analysis

All samples were analyzed using an Acquity UPLC I-class system from Waters hyphenated to a Synapt G2S Q-TOF mass spectrometer, all systems were controlled by Masslynx 4.1 (Waters). Chromatographic separation was performed on an Acquity BEH amide column (2.1 × 100 mm, 1.7 µM particle size, Waters) with 5 mM ammonium formate and 0.025% formic acid in 95:5 ACN:water (*v*/*v*) and in 40:60 ACN:water (*v*/*v*) as mobile phase A and mobile phase B respectively. An elution gradient from 100% mobile phase A to 100% mobile phase B was used, in detail, 100% A was kept for 0.5 min in the beginning followed by a non-linear decrease (slope factor 8 in Masslynx) of A to 100% B over 13.5 min. The non-linear gradient was defined as follows C(t) = C_i_ + (C_f_ − C_i_)*X^3^, where X = (t − T_i_)/(T_f_ − T_i_) and C(t) are the composition C in percent at time t, C_i_ the initial mobile phase composition, C_f_ the final mobile phase composition, T_i_ and T_f_ the initial and final times respectively. Thereafter 100% B was kept for 2 min, then the composition was changed back to 100% A (linearly over 1 min) followed by 6 min at 100% A for re-equilibration. The flow rate was set to 0.4 mL min^−1^, the column temperature was set to 40 °C and the injection volume was 5 µL for all analyzes. 

All samples were analyzed in positive ionization in resolution mode, scanning between *m*/*z* 50 and 800 in MS^E^ mode (alternating scans with high and low collision energy respectively) with a scan time of 0.3 s. Data was collected over the entire chromatographic gradient between 0 and 17 min. The source temperature was set to 120 °C, the capillary voltage to 1 kV, the cone voltage to 30 V and the source offset was set to 50 V. Nitrogen was used as desolvation gas at 500 °C and a flow rate of 800 L h^−1^ as well as cone gas with a flow rate of 50 L h^−1^. Leucine enkephalin, *m*/*z* 556.2771, was infused for lock-spray correction (real-time mass correction) in all analyses at a flow rate of 10 µL min^−1^. During analysis of plasma and cell samples the respective QC samples were injected repeatedly in the beginning of each run to condition the system and then every fifth injection throughout the runs. The QC samples injected throughout the runs were used to monitor retention time drift as well as signal intensity drift [25].

### 3.8. Data Analysis and Multivariate Data Analysis

For multivariate data analysis the raw data was converted to NetCDF format using Databridge (Masslynx) and was processed using the R-based program XCMS [26,27,28]. Peak-picking was performed using the centwave function with the ppm setting set to 40 ppm, the peak width between 5 s and 40 s, the prefilter at 1800 counts in 3 consecutive scans and the noise threshold at 2000. Retention time alignment was performed using the obiwarp function with the binsize set to 0.01 and grouping of peaks was performed using the peak density function with binsize set to 0.01 and the bandwidth (bw) to 2. In the grouping of peaks, the minfraction was set to 1, i.e., a peak had to be present in all samples of at least one samples group (QC, PPT or SPE MCX). Finally, the fillpeaks function was used to integrate regions of missing peaks if possible. The resulting data set was filtered to remove all features eluting before 45 s, and all features with a CV (%) > 30% in the QC samples. Principal component analysis (PCA) and orthogonal projection to latent structures—discriminant analysis (OPLS-DA) was used to find discriminating features between the two sample preparations using SIMCA (Umetrics, Umeå, Sweden). 

Targetlynx (Waters) was used to process data and integrate peaks when multivariate data analysis was not employed, the data was then exported to Microsoft Excel for further processing. The post-column infusion data was exported as extracted ion chromatograms to Microsoft excel where the relative intensities as compared to solvent blank injections were calculated.

## 4. Conclusions

Detection sensitivity in HILIC-ESI-MS of endogenous and food-related organic compounds in human blood plasma can be significantly improved by reduction of Na^+^ and K^+^ concentrations using cation exchange SPE. The improved detection sensitivity was achieved through reduced ion suppression from Na^+^/K^+^ as well as reduced adduct formation and cluster formation of analytes. The investigated analytes spanned a wide range of physicochemical properties and substance classes typically investigated in metabolomics applications. Moreover, multivariate data analysis demonstrated to be a useful method for untargeted investigation into which metabolites that gained and lost in signal intensity due to the SPE treatment. Reduced adduct formation and cluster formation was confirmed by multivariate data analysis, and resulted in both improved signal intensity of analytes as well as reduced complexity of the dataset and more straight forward identification of analytes. The linearity of response was improved or unchanged for five out of six investigated ions. This highlights the importance of controlling factors such as ion-suppression and adduct formation, especially in applications where internal standards are not used to control variations in sample composition.

## Figures and Tables

**Table 1:** Summary of results from the calibration curves. Tryptophan and inosine were detected as fragments due to extensive in-source fragmentation which is noted as [F]^+^. * The calibration range contains too few “passed” calibration standards for a linear range to be determined.

Analyte	Concentration Range of Calibration Curve	Expected Concentration in Blood Plasma	R^2^		No. of Standards within ±15% of Nominal Concentration		Concentration Range of “Passed” Standards	
			PPT	MCX	PPT	MCX	PPT	MCX
Guanine [M+H]^+^	18 nM–1.9 µM	0.4 µM	0.98750	0.99475	4	7	(0.18–1.9 µM) *	30 nM–1.9 µM
Inosine [F]^+^	79 nM–5.4 µM	0.2–0.3 µM	0.98940	0.99510	7	5	0.12–5.4 µM	0.28–5.4 µM
Tryptophan [F]^+^	1.1 µM–110 µM	50–60 µM	0.99707	0. 99396	7	7	2.2–110 µM	2.2–110 µM
Valine [M+H]^+^	77 µM–309 µM	200–250 µM	0.60632	0.85888	2	6	(206–309 µM) *	103–309 µM
Tryptophan [M+2Na−H]^+^	1.1 µM–110 µM	50–60 µM	0.98341	0.98519	4	5	(11–110 µM) *	11–110 µM
Valine [M+2Na−H]^+^	77 µM–309 µM	200–250 µM	0.96423	0.95860	8	8	77–309 µM	77–309 µM

## Data Availability

The data presented in this study are available within the article and Supplementary Material.

## Supplementary Materials

The following are available online at https://www.mdpi.com/2218-1989/11/3/170/s1, Figure S1. Summary of results where each elution fraction from plasma samples were collected separately and analyzed, Table S1: Summary of the selected analytes in the standard solutions, Table S2: Summary of investigated analytes in plasma, Table S3: Summary of investigated analytes in cells, Table S4: Summary of results from evaluating protein precipitation prior to SPE, Table S5. Summary of all annotated features with higher intensity in SPE MCX, Table S6. Summary of all annotated features with higher intensity in PPT.

## Appendix A

**Table A1:** Summary of relative peak area ^1^ and CV (%) of all analytes as well as Na^+^ for all tested SPE protocols in standard solution. ^1^ mean of SPE replicates/mean of standard solution replicates expressed as percent (%), ^2^ The analytes were detected as fragment.

	SCX-2		MCX		StrataX-C		WCX Acidic		WCX Basic		Standard Solution
	Rel. int. (%) ^1^	CV (%)	Rel. int. (%) ^1^	CV (%)	Rel. int. (%) ^1^	CV (%)	Rel. int. (%) ^1^	CV (%)	Rel. int. (%) ^1^	CV (%)	CV (%)
Butyrylcarnitine	88.9	4.56	101	3.22	59.9	14.0	119	19.0	157	34.52	16.7
Hypoxanthine	94.2	6.77	102	4.86	101	28.5	50.9	15.3	185	40.56	51.0
Choline	0.070	43.9	0.800	44.2	0.080	19.1	44.6	11.1	123	49.35	34.0
Thiamine [F] ^2^	0.210	4.28	1.10	12.9	0.040	31.2	40.3	3.43	2.98	21.15	11.4
Adenine	95.3	13.7	106	5.42	90.7	18.7	70.8	6.76	164	66.83	40.7
Betaine	120	8.65	132	5.39	65.7	19.2	39.1	4.73	219	10.11	55.9
Phenylalanine [F] ^2^	165	13.5	176	22.3	87.8	29.7	45.0	31.7	444	17.48	44.3
Tryptophan [F] ^2^	119	13.6	161	4.68	118	27.3	45.3	10.1	353	34.54	49.4
Acetylcarnitine	114	6.92	192	4.98	151	41.2	137	8.42	297	40.62	12.0
Proline	336	4.14	537	4.65	296	27.5	62.0	12.4	1278	57.03	17.3
Taurine	329	20.0	383	32.3	311	34.1	18.7	24.9	1467	51.0	43.5
Carnitine	105	22.4	119	7.90	103	33.6	58.2	9.47	144	35.5	34.5
Creatine	92.5	15.3	119	14.3	39.0	44.1	9.62	14.2	269	54.9	106
Arginine	0.180	14.1	11.6	12.3	0.820	61.9	0.540	4.81	184	70.3	206
NADH	13.1	5.82	53.6	3.3	185	14.5	0.350	5.28	636	26.5	216
Sodium, Na+	2.53	14.0	4.29	11.0	1.81	50.2	18.0	7.58	17.5	59.8	76.8

**Table A2:** Summary of relative peak area ^1^ and CV (%) of the investigated analytes in the experiment where pH buffering in all steps of the SPE protocol was tested. ^1^ mean of SPE replicates/mean of PPT replicates expressed as percent (%). ^2^ The analyte was detected as a fragment.

	SPE MCX No pH Control		SPE MCX pH Control		WCX Basic No pH Control		WCX Basic pH Control		PPT
	Rel. int. (%) ^1^	CV %	Rel. int. (%) ^1^	CV %	Rel. int. (%) ^1^	CV %	Rel. int. (%) ^1^	CV %	CV %
Caffeine 0.89 min	75.06	60.24	83.65	26.55	75.79	39.91	83.98	29.66	5.58
Theophylline 1.21 min	62.51	5.47	71.66	14.79	91.01	5.60	96.13	4.82	4.47
Hypoxanthine 4.39 min	113.06	3.30	72.36	15.81	112.73	2.85	117.88	10.31	6.13
Creatinine 4.69 min	118.92	5.50	71.64	23.31	119.93	5.19	130.80	14.93	8.21
Palmitoylcarnitine 5.09 min	96.37	9.51	73.63	17.11	95.10	0.92	155.56	17.19	2.81
Choline 6.01 min	1.74		3.17		5.59		28.18	11.43	5.48
Pantothenic acid ^2^ 7.35 min	95.41	3.35	67.88	3.15	97.59	5.04	64.48	11.43	2.12
Hexanoylcarnitine 7.8 min	95.89	42.41	92.61	28.38	102.74	7.84	131.01	19.49	4.69
1-Methylnicotinamide 8.02 min	0.00	4.15	0.00	22.91	0.00	6.68	12.48	4.92	7.58
Butyrylcarnitine 9.08 min	273.41	1.17	77.49	20.43	278.09	9.84	83.05	13.50	12.63
Oxoproline 9.13 min	311.67	15.80	110.16	38.51	357.32	21.98	77.07	22.53	11.54
Homostachydrine 9.41 min	100.71	38.70	65.39	7.51	115.96	96.24	52.53	87.20	21.20
Propionylcarnitine 9.78 min	210.52	7.09	94.03	18.04	243.53	4.66	71.86	3.49	16.65
Betaine 9.83 min	108.60	6.86	79.86	14.09	112.69	13.49	71.19	2.09	16.92
Methylnicotinic acid 10.16 min	181.86	4.16	80.55	14.61	112.79	12.95	46.43	8.46	9.85
Acetylcarnitine 10.47 min	604.31	3.36	277.47	14.83	784.74	5.22	139.35	10.22	11.28
Acetylcholine 10.78 min	2279.60	5.52	247.01	15.20	1183.32	8.53	142.26	20.24	12.07
Carnitine 10.89 min	279.17	1.59	122.97	9.92	281.05	10.11	73.42	2.48	3.67
Creatine 11.07 min	181.63	10.00	79.86	12.13	178.08	8.59	42.96	15.11	3.10
Arginine 12.5 min	52.15	2.95	28.85	9.38	39.26	16.09	22.32	20.15	3.68
