# Supplementary material for: Improved Sensitivity in Hydrophilic Interaction Liquid Chromatography-Electrospray-Mass Spectrometry after Removal of Sodium and Potassium Ions from Biological Samples
Source: Metabolites. 2021 Mar 15;11(3):170. doi: 10.3390/metabo11030170 (PMC7999259; doi:10.3390/metabo11030170)
Supplement: Supplementary file 1 [file metabolites-11-00170-s001.zip › Supplementary information_SPE_to_remove_alkaliions.pdf]

Supplementary information - Improved sensitivity in hydrophilic interaction liquid chromatography-electrospray-mass spectrometry after removal of sodium and potassium ions from biological samples

Contents:

- Table S1 – Summary of the selected analytes in the standard solutions
- Table S2 – Summary of selected analytes in plasma that was investigated
- Table S3 – Summary of selected analytes in the cells samples that was investigated
- Table S4- Summary of results from evaluating protein precipitation prior to SPE
- Figure S1 – Summary of results where each elution fraction from plasma samples were collected separately and analyzed
- Table S5- Summary of all annotated features with higher intensity in SPE MCX
- Table S6 – Summary of all annotated features with higher intensity in PPT

Table S 1. Selected analytes in the standard solutions for screening of solid phases

Analyte	*Elemental composition*	*Mw*	*Log p (XlogP3)*	*pKa (acidic)*	*pKa (basic)*	*Retention time (min)*
Adenine	$C_5H_5N_5$	135.13	-0.1	10.29	3.64	3.51
Hypoxanthine	$C_5H_4N_4O$	136.11	-0.5			3.52
Choline	$C_5H_{14}NO^+$	104.17	-0.4	13.97		4.99
Butyrylcarnitine	$C_{11}H_{21}NO_4$	231.29	1.2	4.27		8.38
Betaine	$C_5H_{11}NO_2$	117.15	0.5	2.26		8.82
Phenylalanine [F][2]	$C_9H_{11}NO_2$	165.19	-1.5	2.47	9.45	9.05
Tryptophan [F][2]	$C_{11}H_{12}N_2O_2$	204.22	-1.1	2.54	9.4	9.14
Thiamine [F][2]	$C^{12}H_{17}N_4OS^+$	265.36	1		5.54	9.21
Acetylcarnitine	$C_9H_{17}NO_4$	203.24	0.4	4.09		9.68
Proline	$C_5H_9NO_2$	115.13	-2.5	1.94	11.33	9.79
Taurine	$C_2H_7NO_3S$	125.15	-4.1	-1.5 (sulfonic acid)	9.34	9.87
Carnitine	$C_7H_{15}NO_3$	161.2	-0.2	4.2		10.46
Creatine	$C_4H_9N_3O_2$	131.13	-1.2	3.5	12.43	10.68
Arginine	$C_6H_{14}N_4O_2$	174.20	-4.2	2.41	12.41	12.28
NADH	$C_{21}H_{29}N_7O_{14}P_2$	665.4	-5.7	1.85	4.01	12.37

Table S2. Summary of the selected analytes that was investigate din the plasma

	Elemental composition	*Mw*	*Log p (XlogP3)*	*pKa (acidic)*	*pKa (basic)*	*Retention time (min)*
Caffeine	$C_8H_{10}N_4O_2$	194.19	-0.1			0.86
Theophylline	$C_7H_8N_4O_2$	180.16	0			1.08
Hypoxanthine	$C_5H_4N_4O$	136.11	-0.5			3.52
Creatinine	$C_4H_7N_3O$	113.12	-1.8	9.21	4.96	3.65
Palmitoylcarnitine	$C_{23}H_{45}NO_4$	399.60	7.7	4.22		4.46
Choline	$C_5H_{14}NO^+$	104.17	-0.4	13.97		4.99
Pantothenic acid	$C_9H_{17}NO_5$	219.23	-1.1	4.35		6.09
1-methylnicotinamide	$C_7H_9N_2O^+$	137.16	-0.1	12.24		7.12
Hexanoylcarnitine	$C_{13}H_{25}NO_4$	259.34	2.3	4.22		7.14
Valerylcarnitine	$C_{12}H_{23}NO_4$	245.32	1.8	4.34		7.73
Oxoproline	$C_5H_7NO_3$	129.11	-0.8	3.61		8.29
Butyrylcarnitine	$C_{11}H_{21}NO_4$	231.29	1.2	4.27		8.38
Homostachydrine	$C_8H_{15}NO_2$	157.21	1.4	2.39		8.48
Prolinebetaine	$C_7H_{13}NO_2$	142.18	1	2.26		8.76
Betaine	$C_5H_{11}NO_2$	117.15	0.5	2.26		8.82
Propionylcarnitine	$C_{10}H_{19}NO_4$	217.26	0.9	4.19		9.02
Phenylalanine	$C_9H_{11}NO_2$	165.19	-1.5	2.47	9.45	9.05
Leucine	$C_6H_{13}NO_2$	131.17	-1.5	2.79	9.52	9.10
Tryptophan	$C_{11}H_{12}N_2O_2$	204.22	-1.1	2.54	9.4	9.14
Isoleucine	$C_6H_{13}NO_2$	131.17	-1.7	2.79	9.59	9.36
Acetylcarnitine	$C_9H_{17}NO_4$	203.24	0.4	4.09		9.68
Proline	$C_5H_9NO_2$	115.13	-2.5	1.94	11.33	9.79
Taurine	$C_2H_7NO_3S$	125.15	-4.1	-1.5 (sulfonic acid)	9.34	9.87
Tyrosine	$C_9H_{11}NO_3$	181.19	-2.3	2	9.19	10.01
3-Dehydroxycarnitine	$C_7H_{15}NO_2$	145.20	0.8	4.46		10.38
L-carnitine	$C_7H_{15}NO_3$	161.2	-0.2	4.2		10.46
Creatine	$C_4H_9N_3O_2$	131.13	-1.2	3.5	12.43	10.68
Arginine	$C_6H_{14}N_4O_2$	174.20	-4.2	2.41	12.41	12.28

Table S3. Summary of the selected analytes that was investigate in the cells samples.

	Elemental composition	*Mw*	*Log p (XlogP3)*	*pKa (acidic)*	*pKa (basic)*	*Retention time (min)*
Niacinamide	$C_6H_6N_2O$	122.12	-0.4			1.05
Adenine	$C_5H_5N_5$	135.13	-0.1	10.29	3.64	3.51
Hypoxanthine	$C_5H_4N_4O$	136.11	-0.5			3.52
Choline	$C_5H_{14}NO^+$	104.17	-0.4	13.97		4.99
Pantothenic acid	$C_9H_{17}NO_5$	219.23	-1.1	4.35		6.09
Inosine	$C_{10}H_{12}N_4O_5$	268.23	-1.3	6.94		6.94
1-methylnicotinamide	$C_7H_9N_2O^+$	137.16	-0.1	12.24		7.12
Hexanoylcarnitine	$C_{13}H_{25}NO_4$	259.34	2.3	4.22		7.14
Guanine	$C_5H_5N_5O$	151.13	-1.1	8.95		7.91
Betaine	$C_5H_{11}NO_2$	117.15	0.5	2.26		8.82
Isoleucine	$C_6H_{13}NO_2$	131.17	-1.7	2.79	9.59	9.36
Methyl-deoxyguanosine	$C_{11}H_{16}N_5O_4^+$	282.28	-1.6			9.83
Acetylcarnitine	$C_9H_{17}NO_4$	203.24	0.4	4.09		9.68
Proline	$C_5H_9NO_2$	115.13	-2.5	1.94	11.33	9.79
Taurine	$C_2H_7NO_3S$	125.15	-4.1	-1.5 (sulfonic acid)	9.34	9.87
Arginine	$C_6H_{14}N_4O_2$	174.20	-4.2	2.41	12.41	12.28

Table S6. Summary of relative peak area[1] and CV (%) of the investigated analytes in the experiment where protein precipitation prior to SPE MCX was evaluated.

Analyte, retention time (min)	*MCX with PPT*		*MCX without PPT*		*PPT*
	Relative intensity (%)	*CV (%)*	*Relative intensity (%)*	*CV (%)*	*CV (%)*
Hypoxanthine 4.02	125.6266472	1.378	110.8799044	3.441	21.952
Creatinine 4.2	117.4299843	2.12	133.807133	3.485	4.744489
Palmitoylcarnitine 5.4	85.9602697	26.69	33.40121224	21.08	29.53563
Choline 5.56	2.641500251	19.27	16.61154504	7.061	26.21871
Pantothenic acid 6.84	85.40492916	8.207	80.93837492	5.336	5.590506
2-methylbutyroylcarnitine 8.27	149.3165296	6.293	125.317237	8.285	5.853454
Oxoproline 8.76	228.2463995	2.677	115.1336233	20.12	6.768046
Butyrylcarnitine 8.8	326.7776176	6.254	304.829507	11.39	4.878658
Homostachydrine 8.93	115.729242	3.322	176.7540209	8.003	5.886134
Proline betaine 9.21	116.14128	1.941	208.7181999	7.591	2.874121
Thiamine 9.22	220.2583361	4.059	108.0585633	27.85	6.794585
Betaine 9.28	119.4600827	5.5	212.8528293	7.144	2.439191
Propionylcarnitine 9.39	293.6652519	10.91	278.6311758	6.534	3.088455
Phenylalanine 9.48	126.6451497	3.006	77.60832642	23.57	2.433404
Tryptophan 9.49	106.2957566	1.798	41.48744984	25.86	2.930751
Methylnicotinamide 9.69	0.400363315	173.2	0.171527667	173.2	3.714952
Methylnicotinic acid 9.69	147.4394847	4.138	257.7782455	14.2	3.328579
Acetylcarnitine 10.05	811.7125802	9.421	819.0594752	7.589	4.143839
Proline 10.11	233.2310097	5.982	320.609619	19.61	6.45092
Taurine 10.14	284.1782996	14.46	339.3882756	23.99	15.05482
Acetylcholine 10.62	997.3195326	10.29	718.4421449	6.944	10.72659
Carnitine 10.69	304.2582519	4.811	363.3005072	8.104	9.008662
Creatine 10.88	165.4677771	15.47	195.2015767	35.94	7.764325
Glycerophosphocholine 11.41	479.1616469	53.13	1303.867205	39.53	28.20472
Leucine 9.57	126.7773962	8.391	102.2020429	23.35	5.597462
Isoleucine 9.78[3]	∞	20.14	∞	23.76	0
Sodium Na+	4.648797421	26.54	12.70694818	19.66	1.758547

[1] *mean of SPE replicates/mean of PPT replicates expressed as percent (%)*

[2]*The analyte was detected as a fragment*

[3]*Isoleucine was only detected in SPE MCX*

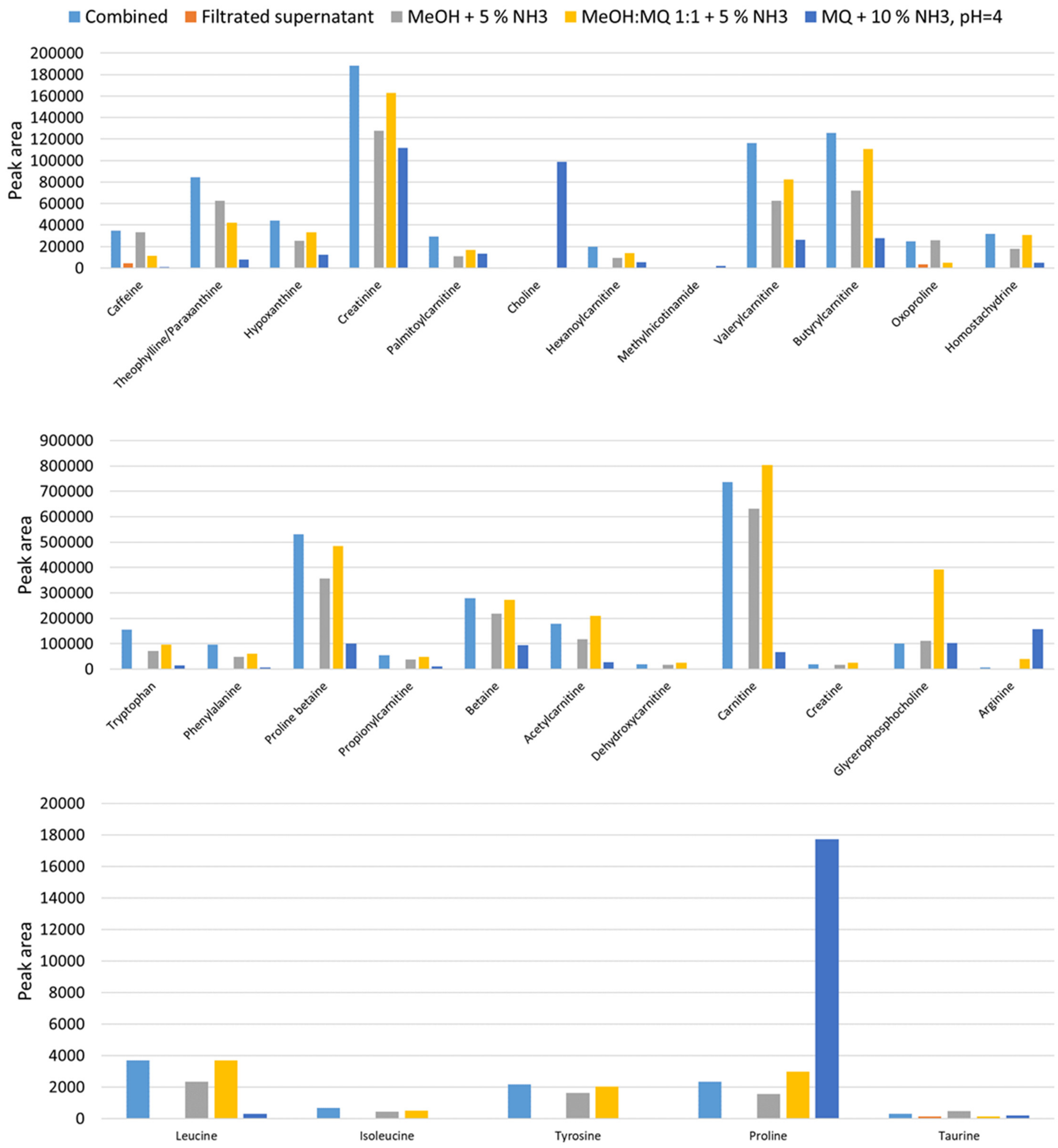

Figure S1. Summary of results for the plasma samples where the filtered supernatant and three different elution fractions were collected separately.

Table S7. Annotated features from the OPLS-DA analysis found in higher intensity in the SPE MCX

Analyte	Adducts	*m/z*	Retention (min)
PEG 12	[M+K] isotope	586.292	1.68
	[M+Na]	569.315	1.68
PEG 13	[M+Na]	613.341	1.76
Creatinine	[M+H]	114.067	3.65
	[F]	86.071	3.65
Palmitoylcarnitine	[M+H]	400.344	4.46
Hexanoylcarnitine	[M+H]	260.186	7.14
Valerylcarnitine	[F]	85.0283	7.74
	[M+H] isotope	247.173	7.73
Oxoproline	[M+H]	130.05	8.29
Butyrylcarnitine	[M+H]	232.153	8.38
	[F]	173.081	8.38
	[F]	85.0283	8.38
Gly Pro Pro Ala*	[M+2Na-H]	385.145	8.44
	[M+H]	341.181	8.44
	[M+H-H2O]	323.17	8.45
	[M+K]	379.137	8.44
	[M+Na]	363.163	8.44
	[M+NH4]	358.207	8.44
Homostachydrine	[M+H]	158.118	8.49
Pro Pro Ala Thr*	[M+H]	385.207	8.51
	[M+2Na-H]	429.171	8.51
	[M+Na]	407.19	8.51
	[M+K]	423.163	8.51
	[M+NH4]	402.234	8.51
Ile Pro Ala Glu*	[M+2Na-H]	473.198	8.57
	[M+H]	429.233	8.57
	[M+K]	467.189	8.57
	[M+Na]	451.216	8.57
	[M+NH4]	446.26	8.57
His Ala Val Phe*	[M+H]	473.259	8.62
	[M+2Na-H]	517.224	8.62
	[M+K]	511.215	8.61
	[M+Na]	495.242	8.62
	[M+NH4]	490.286	8.61
Histidinylproline	[M+2Na-H]	297.093	8.65
	[M+K]	291.084	8.65

	[M+Na]	275.111	8.65
	[M+Na+K-H]	313.066	8.65
Norleucine/Alloisoleucine/Alaninebetaine	[M+H]	132.102	8.70
N-(1-Deoxy-1-fructosyl)valine	[M+H]	280.14	8.73
	[M+H-2H2O]	244.119	8.73
	[M+H-H2O]	262.13	8.73
	[M+Na]	302.122	8.73
Proline betaine	[M+H]	144.103	8.76
	[M+Na]	166.084	8.76
Betaine	[M+H]	118.087	8.82
	[2M+Na]	257.148	8.81
	[F]	58.0649	8.81
	[F]	59.0726	8.82
	[M+Na]	140.069	8.82
Propionylcarnitine	[M+H]	218.139	9.03
	[F]	60.0805	9.03
	[F]	85.0283	9.03
	[F]	159.065	9.03
Ile Ile Phe Arg*	[M+2Na-H]	592.322	9.05
	[M+Na]	570.341	9.07
Phenylalanine	[M+H]	166.086	9.05
	[F]	120.081	9.05
	[F]	149.06	9.05
	[F]	77.0385	9.06
	[F]	93.0698	9.06
	[F]	103.054	9.06
	[F]	107.049	9.06
Phenylpropanolamine	[M+H]	152.107	9.12
Tryptophan	[M+H]	205.097	9.15
	[F]	118.066	9.14
	[F]	91.0542	9.14
	[F]	188.071	9.14
	[F]	170.06	9.14
	[F]	146.061	9.14
	[F]	142.065	9.14
	[F]	117.058	9.14
	[F]	159.092	9.14
	[F]	132.081	9.15
	[F]	130.065	9.15
Methionine	[M+2Na-H]	194.022	9.66
Acetylcarnitine	[M+H]	204.124	9.69
	[F]	145.05	9.69
	[F]	85.0287	9.69
Proline	[M+H]	116.071	9.80

	[F]	70.065	9.80
	[M+2Na-H]	160.035	9.79
Taurine	[M+2Na-H]	169.985	9.88
Valine betaine	[M+H]	160.134	9.96
Tyrosine	[M+H] isotope	183.082	10.05
	[F]	95.0493	10.01
	[F]	91.0542	10.01
	[F]	123.044	10.01
	[F]	119.049	10.01
	[F]	136.076	10.01
	[F]	147.044	10.01
	[F]	165.055	10.02
	[M+Na+K-H]	242.017	10.0182
3-dehydroxycarnitine	[M+H]	146.118	10.38
	[F]	87.044	10.38
Carnitine	[M+H]	162.114	10.47
	[F]	102.091	10.47
	[F]	60.0807	10.47
	[F]	85.0284	10.47
	[F]	103.039	10.47
	[M+Na]	184.094	10.46
Creatine	[M+H]	132.077	10.69
	[F]	90.0549	10.69
	[M+2Na-H]	176.04	10.68
Threonine	[M+2Na-H]	164.029	10.92

* Exact order of amino acids not determined

Table S8. Annotated features from the OPLS-DA analysis found in higher intensity in the PPT.

Analyte	Adduct	*m/z*	Retention (min)
Caffeine	[F]	138.066	0.86
	[M+H]	195.089	0.86
Theophylline	[M+H]	181.073	1.10
PEG 6	[M+H]	283.176	1.19
PEG 5	[M+H]	239.149	1.24
PEG 7	[M+K]	365.158	1.37
PEG 10	[M+K]	497.236	1.38
	[M+Na]	481.262	1.38
PEG 8	[M+K]	409.185	1.42
	[M+Na]	393.211	1.42
PEG 9	[M+H]	415.256	1.48
	[M+K]	453.211	1.48
	[M+Na]	437.236	1.48
PEG 11	[M+H]	503.308	1.60
PEG 12	[M+H]	547.334	1.68
Choline	[M+]	104.107	5.00
Dodecanoylcarnitine	[M+H]	344.28	5.17
Dodecenoylcarnitine	[M+H]	342.264	5.25
Decanoylcarnitine	[M+H]	318.254	5.67
Decenoylcarnitine	[M+H]	314.233	5.81
Decadienoylcarnitine	[M+H]	312.217	6.07
Pantothenic acid	[M+H]	220.12	6.09
Octanoylcarnitine	[M+H]	288.218	6.32
	[M+Na]	310.202	6.38
Methylnicotinamide	[M+]	137.072	7.12
Hydroxydecanoylcarnitine	[M+H]	332.244	7.63
K(HCOOK)n		542.729	7.89
		458.769	7.89
		290.847	7.89
		206.885	7.89
		122.924	8.06
		460.767	7.89
		292.844	7.89
		208.883	7.89
		124.923	7.89
Hydroxybutyrylcarnitine	[M+H]	248.15	8.39
	[M-H2O+H]	230.139	8.39
Gly Pro Pro Ala [M+2K-H]	[M+2K-H]	417.092	8.43
	[M+Na+K-H]	401.119	8.44
Homostachydrine	[M+K]	196.073	8.49

Pro Pro Ala Thr	[M+Na+K-H]	445.145	8.51
Gly His Ile Phe	[M+Na+K-H]	533.197	8.61
Betaine	[M+K]	156.042	8.83
Phenylalanine	[M+Na+K-H]	226.024	9.05
Leucine	[M+2Na-H]	176.066	9.16
Tryptophan	[M+2Na-H]	249.062	9.16
Na(HCOONa)n		770.847	9.52
		702.86	9.52
		634.874	9.52
		566.887	9.52
		498.9	9.52
		430.913	9.52
		362.926	9.51
		294.938	9.53
		226.952	9.52
		158.964	9.52
		90.9765	9.22
		90.9771	9.51
		91.98	9.51
		771.85	9.52
		703.863	9.52
		635.876	9.52
		567.89	9.52
		499.903	9.51
		432.917	9.52
		431.916	9.52
		364.929	9.52
		363.929	9.52
		228.955	9.52
		227.954	9.52
		159.967	9.53
Proline	[M+2Na-H+2HCOONa]	296.009	9.79
	[M+2Na-H+4HCOONa]	431.982	9.79
	[M+2Na-H+HCOONa]	228.021	9.79
Valine	[M+2Na-H+4HCOONa]	433.999	9.83
	[M+2Na-H+5HCOONa]	501.986	9.84
Tyrosine	[M+2Na-H+2HCOONa]	362.019	10.01
	[2M+4Na-3H]	451.988	10.02
